# Supplementary material for: Genetic programming of macrophages generates an in vitro model for the human erythroid island niche
Source: Nat Commun. 2019 Feb 20;10:881. doi: 10.1038/s41467-019-08705-0 (PMC6382809; doi:10.1038/s41467-019-08705-0)
Supplement: Supplementary file 1 — Supplementary Information [file 41467_2019_8705_MOESM1_ESM.pdf]

Genetic programming of macrophages generates an *in vitro* model for the human erythroid island niche.

Lopez-Yrigoyen et al

Supplementary Figures and Tables

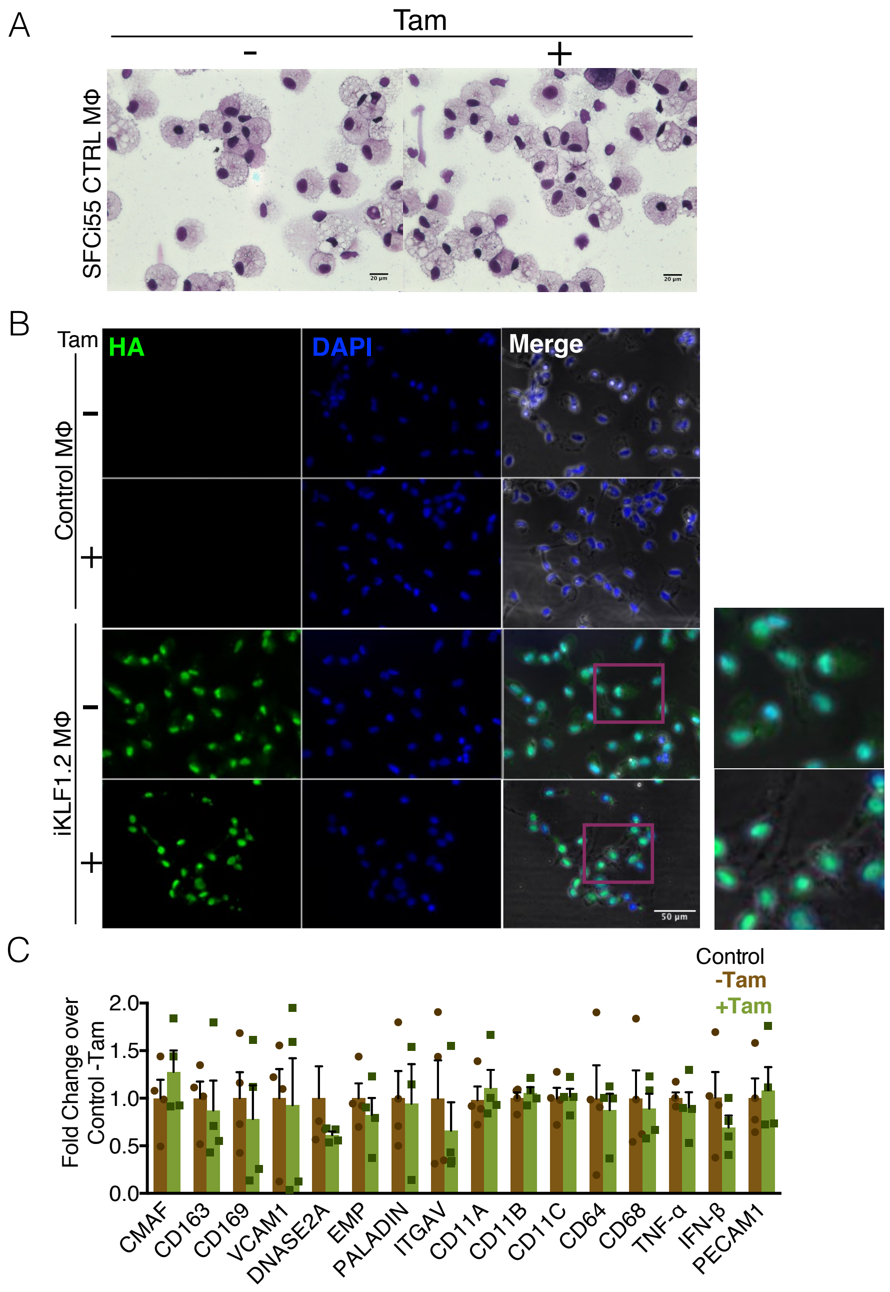

Supplementary Figure 1

A. Kwik-Diff-stained cytospin preparations of control iPSC-DMs in the presence and absence of tamoxifen (Tam)(Scale bar, 20μm).

B. Immunohistochemistry of control iPSC-DM (control Mφ) or iKLF1.2-DMs (iKLF1.2 Mφ) in the presence and absence of tamoxifen using an anti-HA antibody that detects the HA-KLF1-ER^{T2} fusion protein. Insert shows higher magnification demonstrating fusion protein subcellular localisation in cytoplasm in the absence of tamoxifen and in the nucleus after tamoxifen addition (Scale bar, 50μm).

C. Expression of known KLF1 target genes and EI-macrophage related genes in control iPSC-DM in the presence and absence of tamoxifen demonstrating that they are not regulated by tamoxifen alone and that the genes up-regulated in iKLF1.2-DM (Figure 2A) are indeed due to KLF1 activation (n=4 biologically independent samples, non-parametric Wilcoxon Test). [*p<0.05, **p<0.01, ***p<0.001, ****p<0.0001]

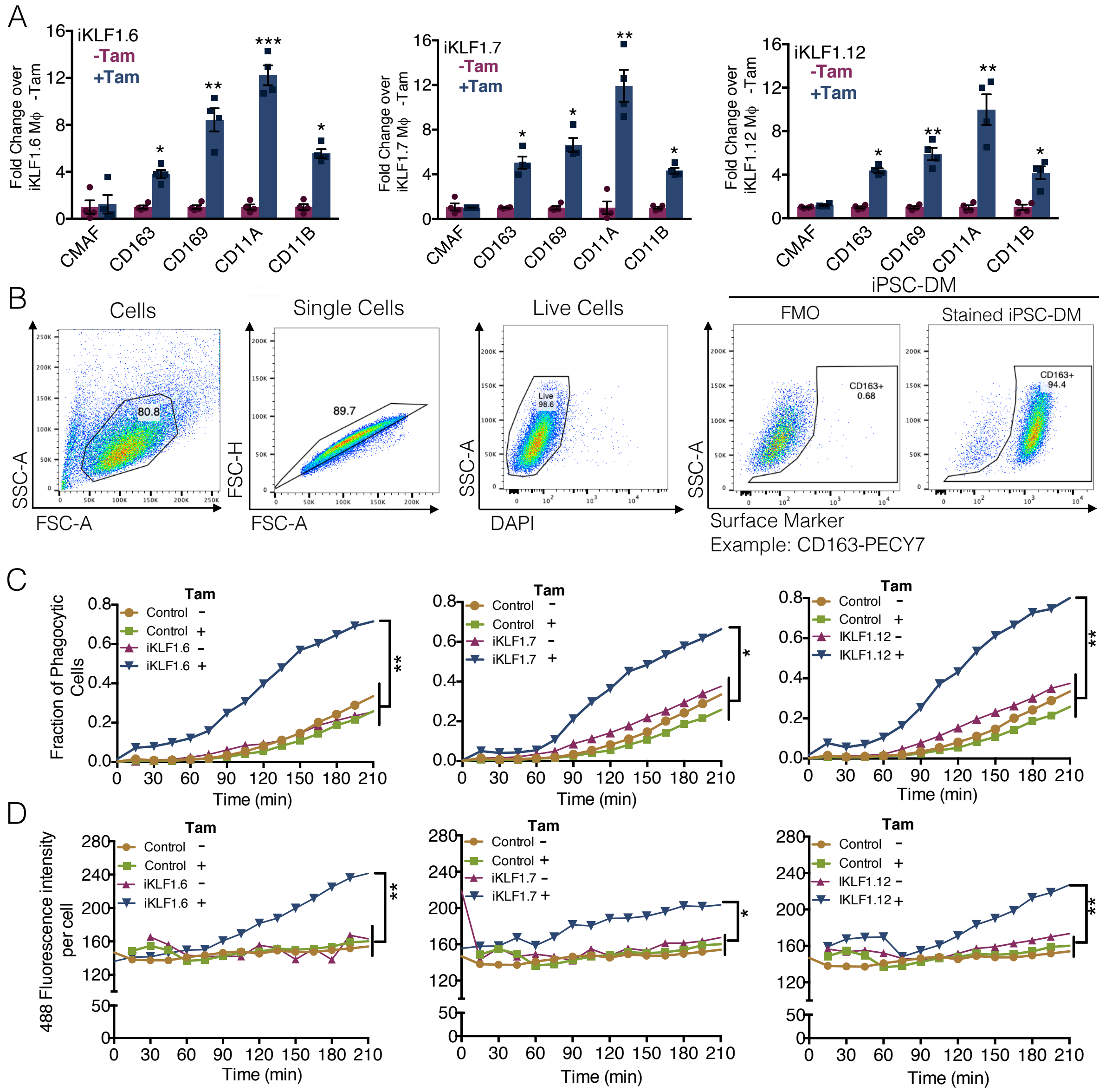

Supplementary Figure 2

A. Quantitative RT-PCR analyses of EI-macrophage related genes in macrophages derived from 3 independently generated iPSC-lines carrying the CAG-KLF1-ER^{T2} transgene in the *AAVS1* locus (named iKLF1.6, iKLF1.7 and iKLF1.12), in the presence and absence of tamoxifen (Tam)(n=4 biologically independent samples, non-parametric Wilcoxon Test).
B. Gating strategy for analysis of iPSC-DM phenotype. Single, live cells were gated, then analysed for the expression of different cell surface markers, using fluorescence minus one (FMO) controls to gate and quantify the proportion of expressing cells. An example of gating (FMO and Control iPSC-DM) for CD163 expression is shown.
C. Phagocytic fraction analyses as measured by the proportion of macrophages containing fluorescent beads from 0 to 210 minutes in control iPSC-DMs and iKLF-DMs (iKLF1.6, iKLF1.7 and iKLF1.12) in the presence or absence of tamoxifen, (n=5 biologically independent samples, two-way ANOVA and Bonferoni post-test).
D. Phagocytic index as calculated by level of green fluorescence per cell in control and iKLF1 iPSC-DM (iKLF1.6, iKLF1.7 and iKLF1.12), in the presence or absence of tamoxifen (n=5 biologically independent samples, two-way ANOVA and Bonferoni post-test). [*p<0.05, **p<0.01, ***p<0.001, ****p<0.0001]

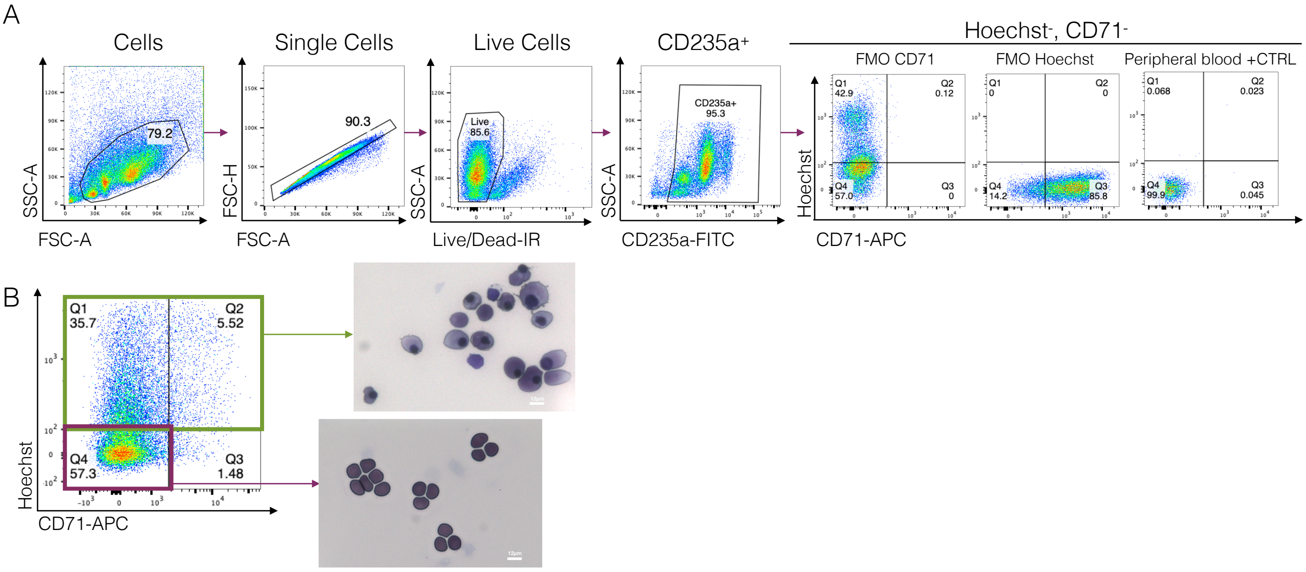

Supplementary Figure 3

A. Gating strategy for analysis of erythroid maturation. Single, live, CD235a^{+} cells were gated, then analysed for the expression of CD71 and Hoechst DNA stain (Fluorescence minus one (FMO) controls are shown). The positive control is peripheral blood showing fully mature, CD71^{-} Hoechst- enucleated RBCs.

B. Kwik-Diff-stained cytospin preparations of single, live cells, CD235a^{+} gated and then sorted based on the expression of CD71 and Hoechst. The population of cells that were identified within the CD71^{-}, Hoechst^{-} population (purple) consisted of only enucleated cells. Cytospin preparations of Hoechst^{+} cell population (green) consisted of nucleated erythroid cells as expected (Scale bar, 12μm).

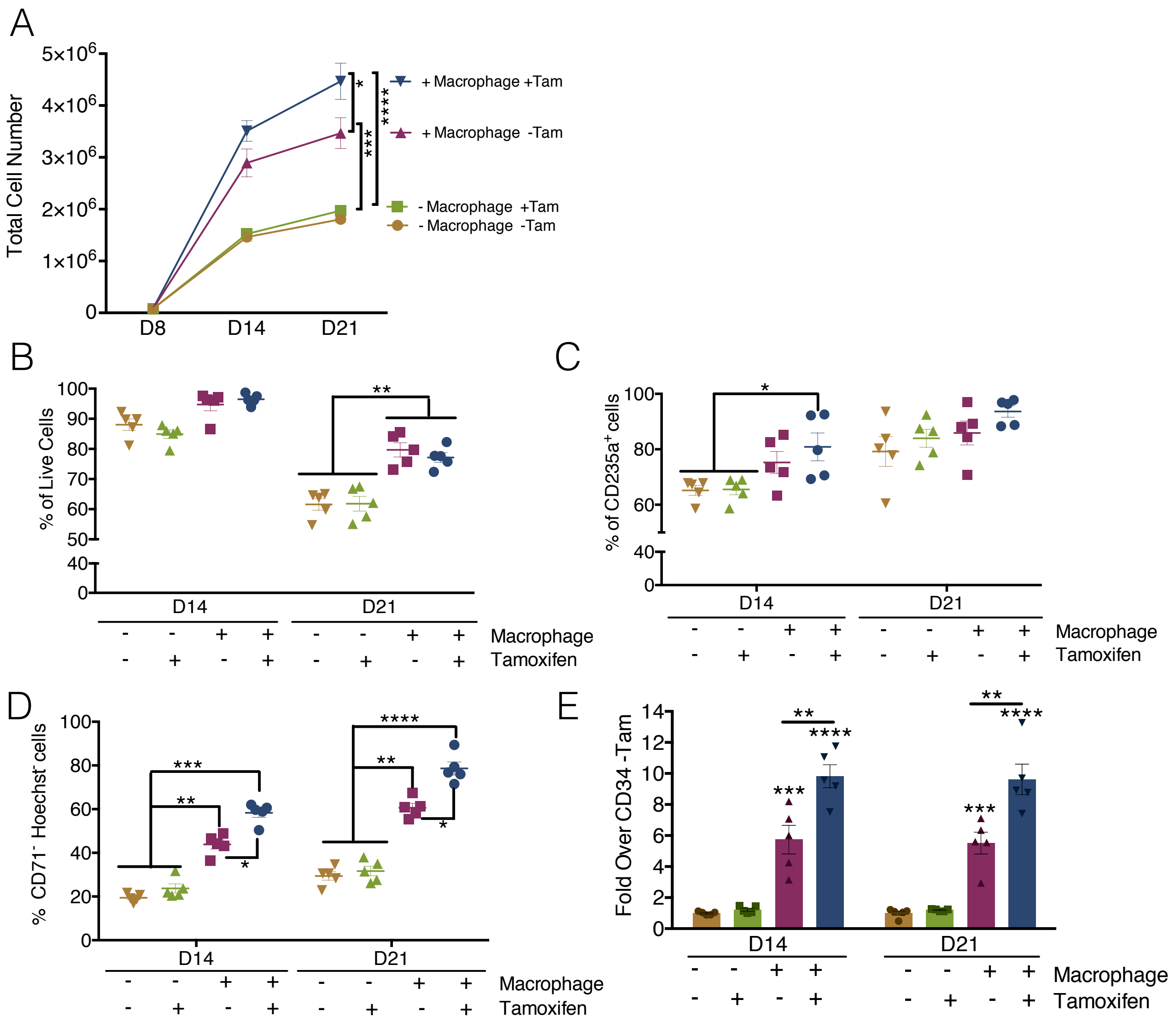

Supplementary Figure 4

A. Total cell counts of UCB CD34$^+$ erythroid cells cultured alone or in co-culture with iKLF1.2-DMs in the presence and absence of tamoxifen (Tam) at day 14 and day 21 (n=5 biologically independent samples, 2-way ANOVA, Tukey's post-test).
B. Percentage of live cells at day 14 and 21 of UCB CD34$^+$ erythroid cells cultured alone or in co-culture with iKLF1.2-DMs in the presence (+) and absence (-) of tamoxifen (n=5 biologically independent samples, 2-way ANOVA with Tukey's post-test).
C. Percentage of live, CD235a-expressing UCB CD34+ erythroid cells cultured alone or in co-culture with iKLF1.2-derived macrophages in the presence and absence of tamoxifen (n=5 biologically independent samples, 2-way ANOVA with Tukey's post-test).
D. Live, CD235a$^+$-gated UCB CD34$^+$derived erythroid cells that are negative for CD71 and Hoechst staining, generated in culture conditions described above (n=5 biologically independent samples; 2-way ANOVA with Tukey's post-test).
E. Fold change of fully mature enucleated UCB CD34$^+$ derived erythroid cells cultured alone or co-cultured in the presence of iKLF1.2-DMs (-/+ tamoxifen) over UCB CD34$^+$ derived erythroid cells cultured alone (- tamoxifen) (n=5 biologically independent samples; 2-way ANOVA with Tukey's post-test) [*p<0.05, **p<0.01, ***p<0.001, ****p<0.0001]

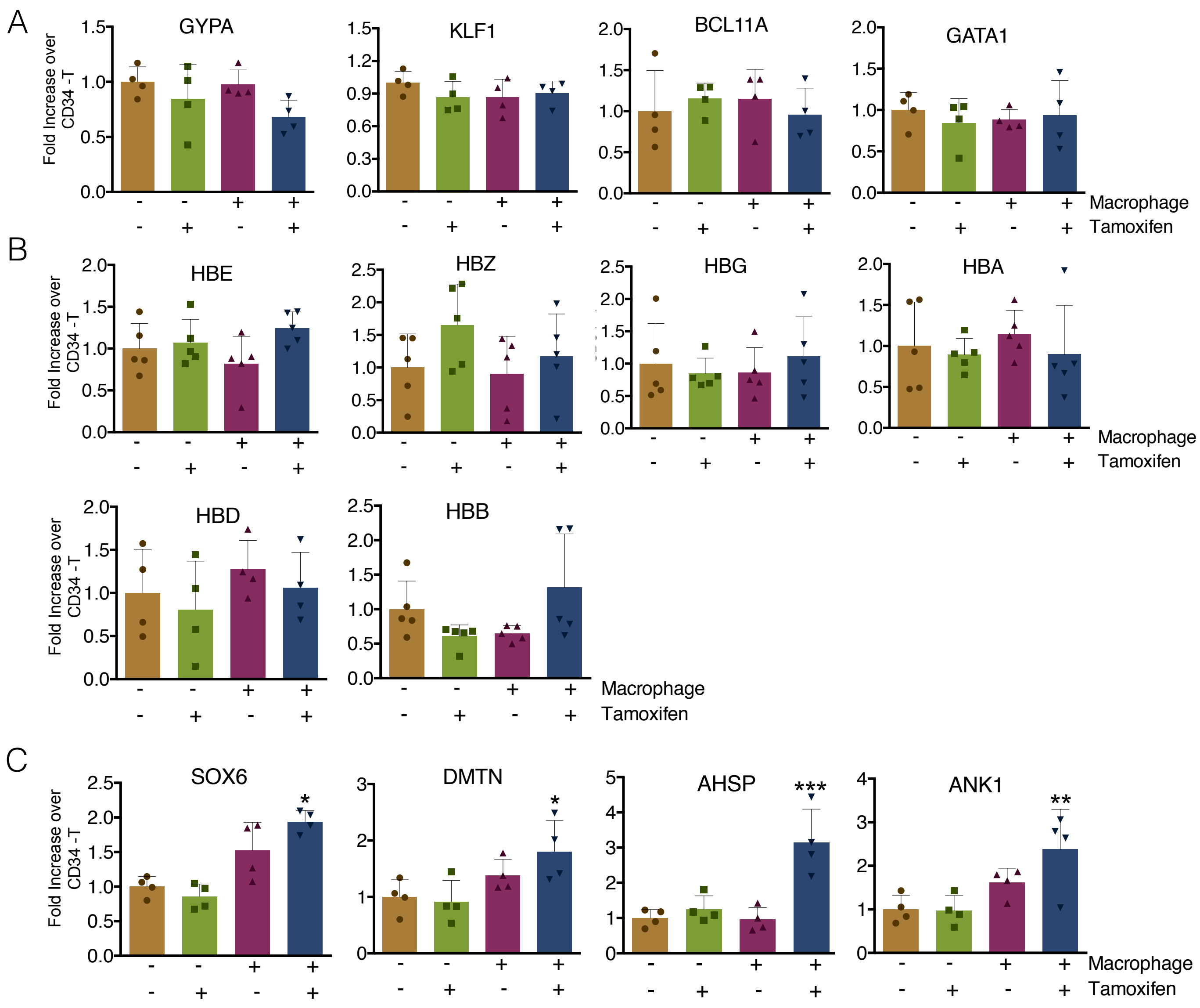

Supplementary Figure 5

UCB CD34+ cells cultured in presence of KLF1-activated macrophages show an enhanced expression of terminal erythroid maturation related genes.

A. Real time PCR analyses of erythroid lineage specification genes; Glycophorin A (encodes CD235a) and transcription factors KLF1, BCL11A and GATA1 in UCB CD34+ cells that were differentiated until day 16 alone or in the presence of iKLF1.2-derived macrophage (-/+ tamoxifen) (n=5 biologically independent samples, One way ANOVA).
B. Real time PCR analyses of globin genes; in UCB CD34+ cells that were differentiated until day 16 alone or in the presence of iKLF1.2-DMs (-/+ tamoxifen) (n=5 biologically independent samples for all, except HBD (n=4), One way ANOVA).
C. Real time PCR analyses of terminal erythroid maturation related genes, SOX6, DMTN, AHSP and ANK1 in UCB CD34+ differentiated until day 16 alone or in the presence of iKLF1.2- DMs (+/- tamoxifen)(n=4 biologically independent samples, One way ANOVA, with Dunnette's post-test). [*p<0.05, **p<0.01, ***p<0.001, ****p<0.0001].

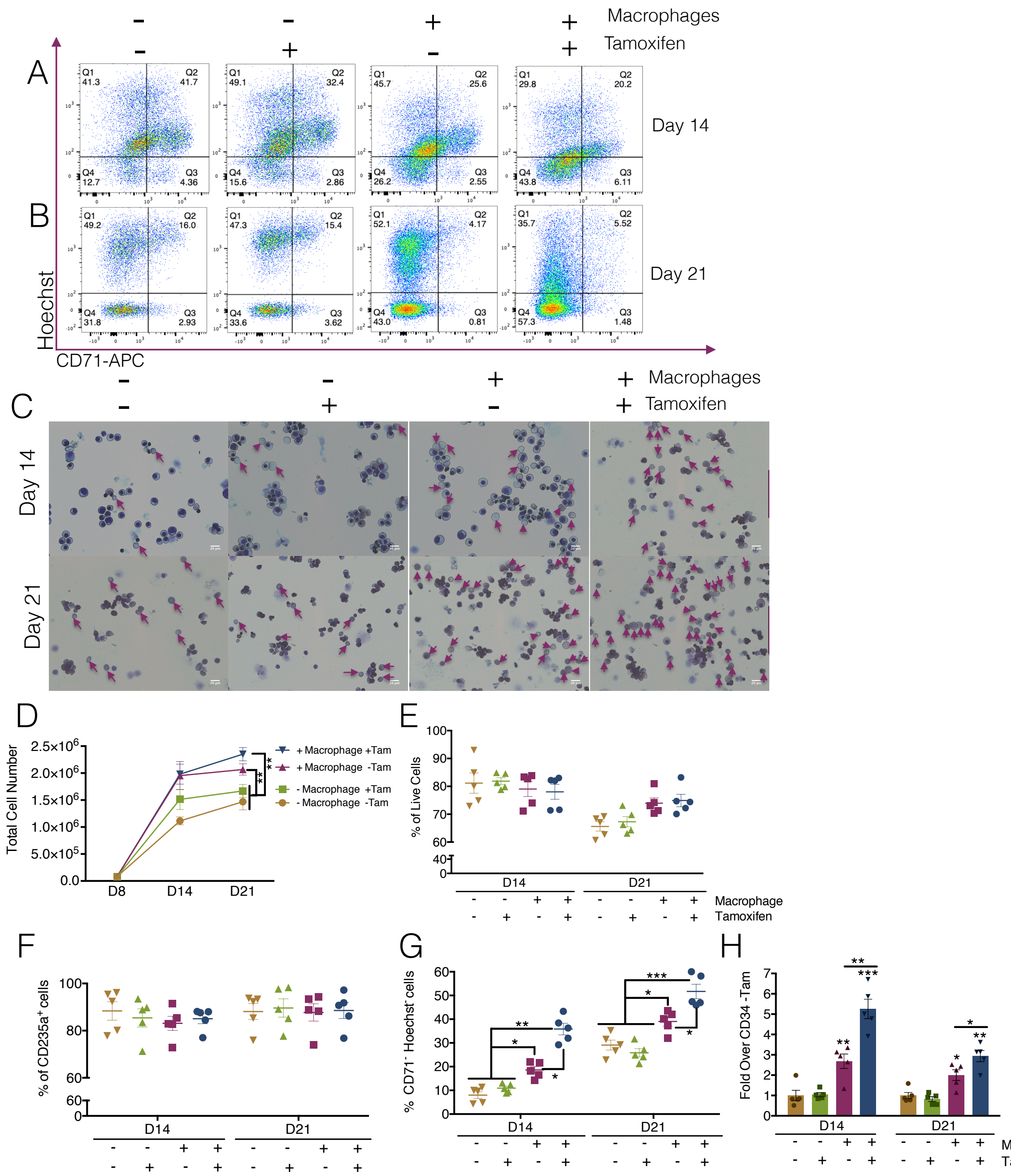

Supplementary Figure 6

Maturation and enucleation of UCB derived CD34^{+} erythroid cells enhanced by KLF1-activated macrophages where contact is prevented in trans-well culture.

A/B. Representative flow cytometry analyses of live, CD235a^{+}-gated UCB-derived erythroid cells cultured alone or in presence of iKLF1.2-DM secreted media in the presence and absence of tamoxifen at day 14 (A) and day 21 (B) stained with anti-CD71 antibody and Hoechst dye (see Supplementary Figure S2 for gating strategy and FMO controls and Figure 4A for quantification).

C. Cytospins of UCB CD34+ cells (from left to right) cultured alone, alone plus tamoxifen, with iKLF1.2-DM secreted media or with secreted media from KLF1-activated macrophages; at day 14 (upper panels) and day 21 (lower panels); arrows point to enucleated cells (scale bar, 20μm).

D. Total cell number counts of UCB CD34^{+} erythroid cells cultured alone or in secreted media from iKLF1.2-DMs in the presence and absence of tamoxifen (Tam) at day 14 and day 21 (n=5 biologically independent samples, 2-way ANOVA, Tukey's post-test).
E. Percentage of live cells at day 14 and 21 of UCB-derived erythroid cells cultured alone or in secreted media from iKLF1.2-DMs in the presence and absence of tamoxifen (n=5 biologically independent samples, 2-way ANOVA with Tukey's post-test).
F. Percentage of live, CD235a^{+} erythroid cells cultured alone or in media secreted from iKLF1.2-DMs in the presence and absence of tamoxifen (n=5 biologically independent samples, 2-way ANOVA with Tukey's post-test).
G. Percentage of live, CD235a^{+}-gated erythroid cells that are negative for CD71 and Hoechst staining generated in the conditions described above (n=5 biologically independent samples; 2-way ANOVA with Tukey's post-test).
H. Fold change in the number of mature enucleated erythroid cells generated in normal media + tamoxifen, and in the presence of secreted media from iKLF1.2-DMs (-/+ tamoxifen) compared to control cultures (CD34-tamoxifen) (n=5 biologically independent samples; 2-way ANOVA with Tukey's post-test)[*p<0.05, **p<0.01, ***p<0.001, ****p<0.0001].

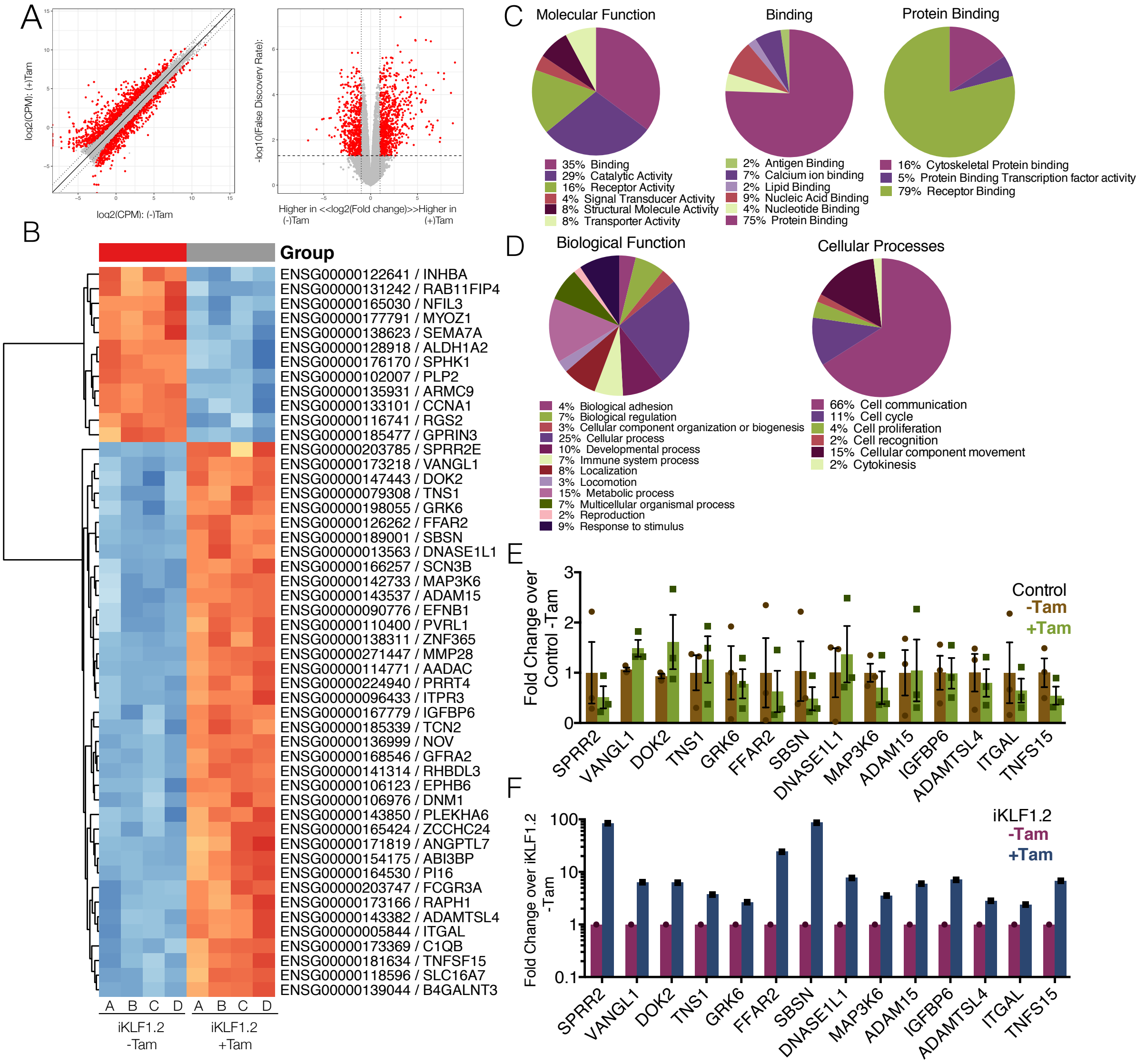

Supplementary Figure 7

Bioinformatic analyses of RNA-sequencing data from iKLF1-derived iPSCs in the presence and absence of tamoxifen.

A. Volcano plots showing up and down regulated genes.
B. Heatmap showing the most differentially expressed genes in iKLF1.2-DMs between the presence and absence of tamoxifen (Tam) (A, B, C and D represent the four biologically independent replicates).
C. Gene ontology analyses of most up-regulated genes according to molecular function, the subset of most enriched category: “binding” and further subset of ‘protein binding’.
D. Gene ontology analyses of the most up-regulated genes according to Biological Function and the subset of the most enriched category: “cellular processes”.
E/F Quantitative-RT-PCR validation of top most differentially expressed genes (most up-regulated genes upon KLF1 activation) in control iPSC-DMs (E) (n=4 biologically independent samples, non-parametric Wilcoxon Test); and in iKLF1.2-DMs (F); in the presence and absence of tamoxifen (Tam). This demonstrates that the increase in gene expression is due to the activation of KLF1 and not a non-specific effect of tamoxifen. [*p<0.05, **p<0.01, ***p<0.001, ****p<0.0001]
Data available at https://www.ncbi.nlm.nih.gov/geo/query/acc.cgi?acc=GSE125150.

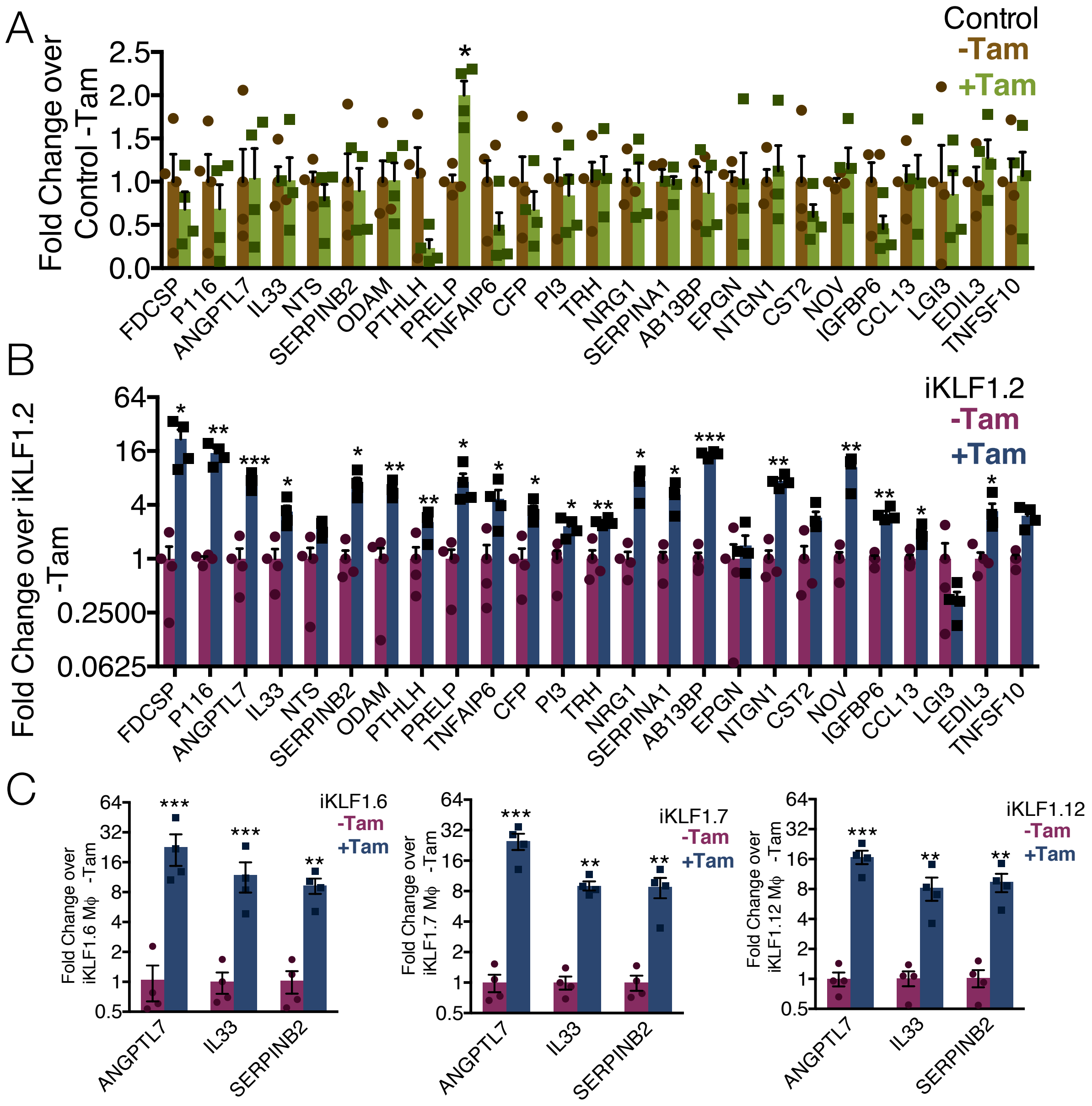

Supplementary Figure 8

A/B Quantitative-RT-PCR validation of the top 25 up-regulated genes that encode secreted factors in control (A) and iKLF1.2-DMs (B) in the presence and absence of tamoxifen. 21 of these were significantly up-regulated in iKLF1.2-DMs upon tamoxifen addition; one gene (PRELP) was up-regulated by tamoxifen nonspecifically and excluded from further analyses. C. Real time PCR analyses of secreted factors ANGPTL7, IL33 and SERPINB2 in macrophages derived from 3 independently generated inducible KLF1 iPSC-lines (left to right: iKLF1.6, iKLF1.7 and iKLF1.12), in the presence and absence of tamoxifen (Tam)(n=4 biologically independent samples, non-parametric Wilcoxon Test). [*p<0.05, **p<0.01, ***p<0.001, ****p<0.0001]

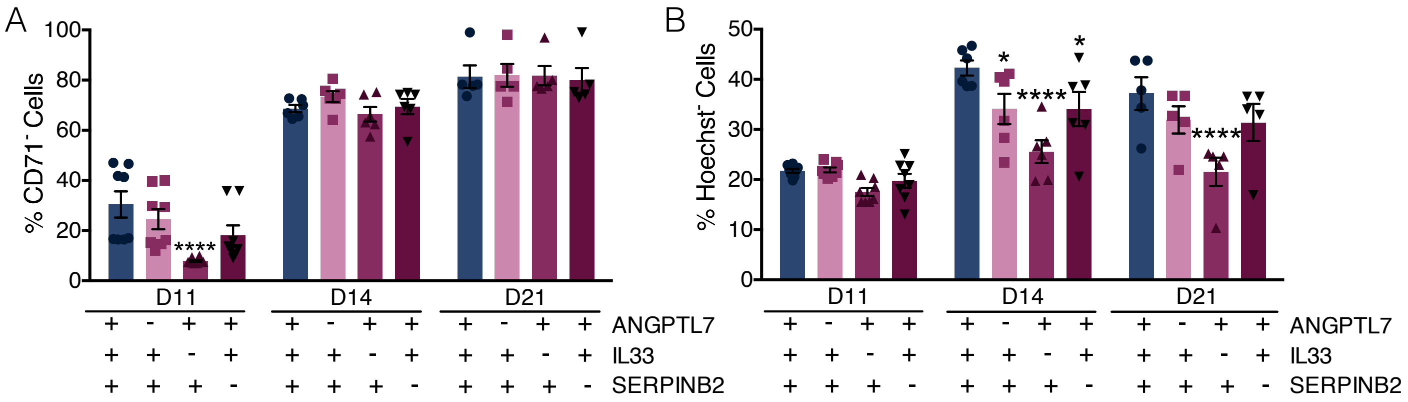

Supplementary Figure 9

A. IL33 removal results in a delay in maturation (in terms of CD71 expression) of $CD34^+$ erythroid cells at day 11: Flow cytometry analyses of live CD235a+ cells looking at CD71 expression of UBC-CD34+ cells that were differentiated in the presence of three secreted factors, or the 3 minus 1 combination (n=5 biologically independent samples biologically independent samples; 2 way ANOVA with Dunnette's post-test).

B. IL33 removal results in a decrease in the percentage of enucleated cells ($Hoechst^-$) at day 14 and 21: Flow cytometry analyses of live $CD235a^+$ cells in UBC-CD34+ cells that were differentiated in the presence of three secreted factors, or their 3 minus 1 combination (n=5 biologically independent samples; 2 way ANOVA with Dunnette's post-test).

A

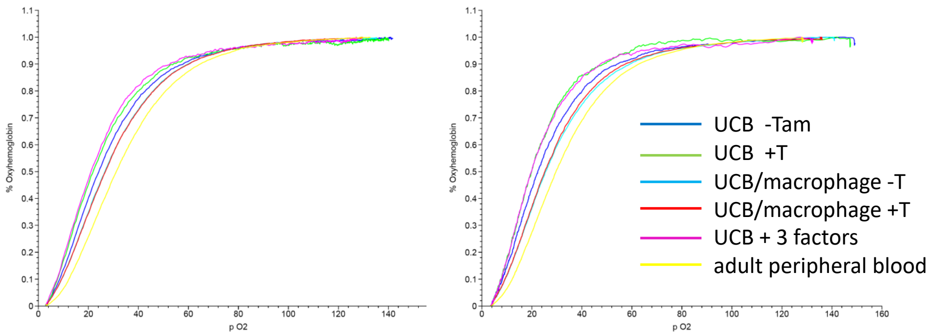

B

Culture conditions	p50 (oxy)	p50 (deox)
UCB - T	24.09/28.31	21.91/22.61
UCB +T	21.82/27.04	19.72/22.97
UCB/macrophage -T	25.55/31.10	25.34/27.28
UCB/macrophage +T	26.78/31.49	25.00/26.52
UCB + 3 factors	21.16	19.56/22.63
Adult peripheral blood	31.16/31.4	29.51/25.56

Supplementary Figure 10

A. Oxygen-haemoglobin association (left) and dissociation (right) curves of differentiated, UCB-derived erythroid cells cultured for 21 days in control conditions or in co-culture with iKLF1.2-DMs in the presence or absence of tamoxifen (T) or in the presence of ANGPTL7, IL33 and SERPINB2. Adult peripheral blood is shown as a control.
B. p50 oxygen tension from two technical replicates of each sample. Although these data are derived from one biological replicate, it is interesting to note that in all conditions where cultured RBCs were generated in the presence of macrophages had a p50 closer to adult peripheral blood compared to cultured RBCs in absence of macrophages.

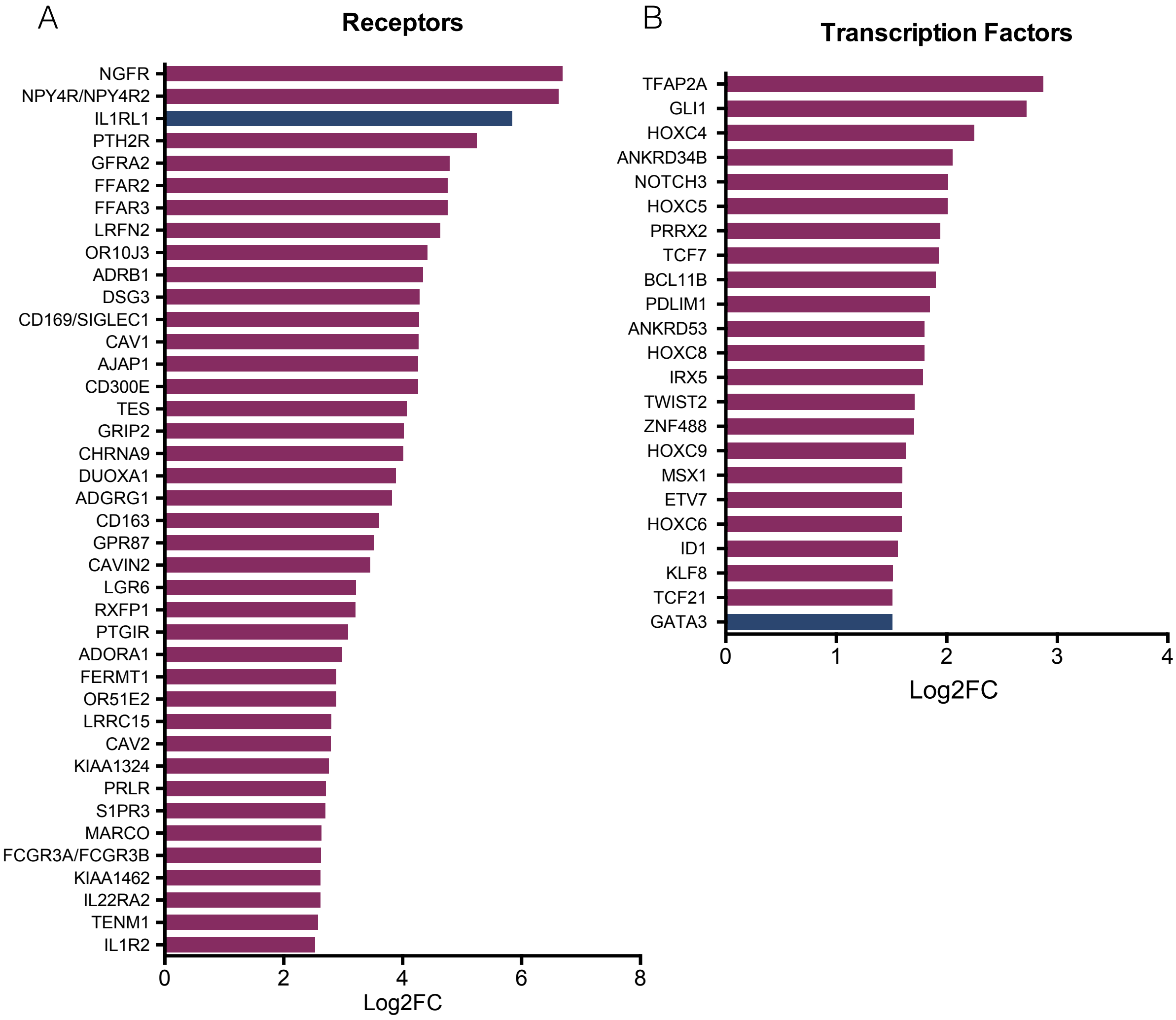

Supplementary Figure 11

A/B. Top most up-regulated genes encoding cell receptors (A) and transcription Factors (B) upon KLF1 activation in iKLF1.2-DMs, categorised by descending Log2 Fold change in expression ($p<0.05$). GATA3 and IL1R1 (green bars) are IL33 target genes.

Supplementary Table 1

Antibodies and dyes used for Flow Cytometry analyses

Cell Surface Marker	Conjugated Fluorochrome	Manufacturer	Catalogue Number	Dilution
**25F9**	E-FLUOR-660	Ebioscience	15599866	1:20
**CCR5**	PE	Biolegend	313708	1:100
**CD163**	PE-CY7	Biolegend	333614	1:25
**CD169**	APC	Biolegend	346007	1:25
**CD206**	PE	Biolegend	321106	1:100
**IgG APC**	APC	Biolegend	400128	1:100
**IgG PE**	PE	Biolegend	400112	1:100
**IgG PE-CY7**	PE-CY7	Biolegend	400125	1:100
**CD235a**	FITC	Ebioscience	11-9987-82	1:100
**CD71**	APC	Ebioscience	17-0719-42	1:200
**Hoechst**	-	Molecular Probes	R37605	1:20
**Live/Dead Near IR Stain**	-	Molecular Probes	L10119	1:100 from stock solution (50µl)

Supplementary Table 2

Primers used for qPCR analyses

Gene	Forward Primer	Reverse Primer	Efficiency
**AADAC**	TGCAGGAGGGAATTTAGCTG	TTGATCTTGACATCTGGGTCAT	1.85
**ADAM15**	CCCTGAGGATCAAGTTGGAG	CCTGGGACCAACTCCCTATT	2.11
**ADAMTSL4**	CACTGAAGCCCCTCTGCTT	CACCTGTGCTGTCTCCTGAC	2.072
**AHSP**	CCTCAAGAGAGTGTGGGTGAGAC	TCCTTATTGGCCTTAAGAAGAGC	1.992
**ANGPTL7**	AACAACCAAATTGACATCATGC	GGTAGAGGGAAGAGCAGTCG	1.89
**ANK1**	TTCACCCAAGTGGTGCAG	CTCATCCGTGAATTGCTCCT	2.011
**B2M**	TTCTGGCCTGGAGGCTATC	TCAGGAAATTTGACTTTCCATTC	2.07
**BCL11A**	CCAAACAGGAACACATAGCAGA	GAGCTCCATGTGCAGAACG	1.893
**CD11A**	CCAGAAGTGAGAGCAGGCTATT	GAGGCCAGCAACGAAGTCT	2.077
**CD11B**	GGCATCCGCAAAGTGGTA	GGATCTTAAAGGCATTCTTTCG	2.014
**CD11C**	CGTGGTCCAGTATGCCAAC	TGGTTGGCAGCTGTTATCTTT	1.879
**CD163**	GAAGATGCTGGCGTGACA	GCTGCCTCCACCTCTAAG	2.006
**CD169**	GGATCATCCAACACCTCACTC	GGTGATGGTGACACCTGGA	2.081
**CD64**	GACCCCATACAGCTGGAAATC	ACCTCAAGGCCAGAGGTTCT	1.993
**CD68**	GTCCACCTCGACCTGCTCT	CACTGGGGCAGGAGAAACT	2.111
**DMTN**	CTCCCAGCTGCTCTTCTACAG	CTTCTGCAGCCGTTCCAT	1.988
**DNASE1L1**	CCTGGATTCCTGGGGTGT	AGGAAGAGGAGTGCAGTTGG	2.049
**DNASE2A**	CAAGGCTCAGGACTCTTCCAT	CACTGTGGACCAGCCAGAA	1.889
**DOK2**	CTGCGGGGGTCCTATACC	AACCTGTAGGGCCAGTCGTA	2
**EFNB1**	TGAGCAGCTGACTACCAGCA	TGTGTGGCCATCTTGACAGT	2.014
**EMP/MAEA**	AAGAGACTGGACGCTGTGAGA	GTCCAGCTGGCTCCCTTC	2.059
**EPHB6**	AATGCTCCCTGCTCACCAT	AACTGGCCCGGTAGAAGC	2.05
**FFAR2**	GAGGTCGCCTGGGTTACAC	GAATCTGGATGGGAGCCTTC	1.888
**GAPDH**	AGCCACATCGCTCAGACAC	GCCCAATACGACCAAATCC	2.05
**GATA1**	CTCCTGACCCTGGGACCT	AGTCAGGGCCCCCATAAG	2.001
**GATA3**	CTCATTAAGCCCAAGCGAAG	TCTGACAGTTCGCACAGGAC	2.07
**GRK6**	CTGCCTCCTGTACGAGATGAT	CCTCCTCCCGCTTGATCT	1.991
**GYPA**	GACACATATGCAGCCACTCCT	ATGATGGGCAAGTTGTACCC	1.997
**HBA**	GACCCGGTCAACTTCAAGC	AGAAGCCAGGAACTTGTCCA	1.956
**HBB**	ACGTGGATGAAGTTGGTGGT	ACCTCTGGGTCCAAGGGTAG	2.008
**HBD**	CAAAGTGAACGTGGATGCAG	TCCAAGGGTAGACCACCAGT	2.029
**HBE**	GGAGAAGGCTGCCGTCACTA	TTCACCRCCAGCCTCTTCCA	2.016
**HBG**	TGGATCCTGAGAACTTCAAGC	GCCACTGCAGTCACCATCT	2.053
**HBZ**	GTGTCCATGTGGGCCAAG	GAAGTGCGGGAAGTAGGTCTT	1.913
**IGFBP6**	CAGATGGCAATGGAAGCTC	TCTATCCCCCAGCTTTAGCC	1.89
**IL1R1**	CTTGTCTGGGGACCCTGTAG	TTTGCCTGCAGCACCTCT	1.99

**IL33**	CCACCAAAAGGCCTTCACT	AAGGCAAAGCACTCCACAGT	2.08
**INF-β**	CGACACTGTTCGTGTTGTCA	GAAGCACAACAGGAGAGCAA	1.885
**ITGA4**	GATGAAAATGAGCCTGAAACG	GCCATACTATTGCCAGTGTTGA	1.887
**ITGAL**	ACTTTGGATACCGCGTCCT	GGCTTCCTGTGCTGTTCC	2.061
**ITGAV**	AAGCTGAGCTCATCGTTTCC	GCACAGGAAAGTCTTGCTAAGG	2.046
**ITPR3**	TGCAGACCTCACTGTCGTGT	GGAGCAAGATCGTCCATCA	1.987
**KLF1**	ACACCAAGAGCTCCCACCT	GTAGTGGCGGGTCAGCTC	1.895
**MAF**	GTACAAGGAGAAATACGAGAAG	TATGAAAAACTCGGGAGA	2.05
**MAP3K6**	CGAGGTCAGAGGAGCTGAGTA	CAGCTGCACCATCAGAGG	2.17
**MMP28**	TGGGACTGGACTTTGTCAGTAA	CCTCAAGCCAAGAGGTCCTA	1.98
**NEURL1**	TCGGCTGTTATGCTGTTCTTC	AGCACCAGCTCGCTATCAA	2.11
**NEURL1B**	TTCTGTGGGAGAGAAAGAATCC	GCTGTCTGCATCGTGGAGT	1.88
**PALLADIN**	TGAACCACACCAGGAGAACA	TCCGAAAGTGCTGCATAGC	2.107
**PECAM**	GCAACACAGTCCAGATAGTCGT	GACCTCAAACTGGGCATCAT	2.117
**PLAUR**	CTTGTGGGAAGAAGGAGAAGAG	GATCTTCAAGCCAGTCCGATAG	1.91
**PRRT4**	CGTCCTGTTTGCTGCTACTG	AGGCCTCACTTTGAGGTACG	2.007
**PVRL1**	CTTCACCGATGGCACTATCC	CGCAGATGTAGACACCCTCA	1.959
**SBSN FW**	CTGAATGGCAACCATCAAAG	GAGGCCCCAGAGGCTAAC	2.056
**SCN3B**	TTGGTTCTCTTTCTAGTTGGTTTGT	CCCAGGAAGGAGTTTAATTGC	1.983
**SERPINB2**	ACCTCATACAGATTCTGCAAAGATT	GCCACACAAAGATCCTCCAT	2.05
**SOX6**	GGACTCAGCCCTTTACAGCTC	TGGCTGTGGAGTTGATGG	1.979
**SPRR2E**	TGGTACTTGAGCACTGATCTGC	TGCACTGCTGCTGTTGATAA	1.99
**TNF-α**	CAGCCTCTTCTCCTTCCTGAT	GCCAGAGGGCTGATTAGAGA	2.02
**TNFSF15**	CTCTGCCTCCCAGGTTCAC	GGAGCCTGTAGTCCCAGCTA	1.96
**TNS1**	GATTGAACAGTCCATCGAAACA	TTGTGTAGTGGGGCAGCAG	2.031
**VANGL1**	CCCAGCATTGTTTCCATAGG	CACTGCCCTCTCTCCCTGT	2.07
**VCAM1**	TGCACAGTGACTTGTGGACAT	CCACTCATCTCGATTTCTGGA	1.97
**ZNF365**	AAGGAGGTTCAAGGGAAAGC	GCCAGTTCTACCCGCTGTAA	2.017
**β-ACTIN**	CCAACCGCGAGAAGATGA	CCAGAGGCGTACAGGGATAG	2.06
