# Supplementary material for: Genetic programming of macrophages generates an in vitro model for the human erythroid island niche
Source: Nat Commun. 2019 Feb 20;10:881. doi: 10.1038/s41467-019-08705-0 (PMC6382809; doi:10.1038/s41467-019-08705-0)
Supplement: Supplementary file 3 — Reporting Summary [file 41467_2019_8705_MOESM3_ESM.pdf]

nature research

Corresponding author(s): NCOMMS-18-21000B

Last updated by author(s): Jan 22, 2019

Reporting Summary

Nature Research wishes to improve the reproducibility of the work that we publish. This form provides structure for consistency and transparency in reporting. For further information on Nature Research policies, see Authors & Referees and the Editorial Policy Checklist.

Statistics

For all statistical analyses, confirm that the following items are present in the figure legend, table legend, main text, or Methods section.

n/a	Confirmed	
☐	☒	The exact sample size (*n*) for each experimental group/condition, given as a discrete number and unit of measurement
☐	☒	A statement on whether measurements were taken from distinct samples or whether the same sample was measured repeatedly
☐	☒	The statistical test(s) used AND whether they are one- or two-sided<br>*Only common tests should be described solely by name; describe more complex techniques in the Methods section.*
☐	☒	A description of all covariates tested
☐	☒	A description of any assumptions or corrections, such as tests of normality and adjustment for multiple comparisons
☐	☒	A full description of the statistical parameters including central tendency (e.g. means) or other basic estimates (e.g. regression coefficient) AND variation (e.g. standard deviation) or associated estimates of uncertainty (e.g. confidence intervals)
☐	☒	For null hypothesis testing, the test statistic (e.g. *F*, *t*, *r*) with confidence intervals, effect sizes, degrees of freedom and *P* value noted<br>*Give P values as exact values whenever suitable.*
☒	☐	For Bayesian analysis, information on the choice of priors and Markov chain Monte Carlo settings
☒	☐	For hierarchical and complex designs, identification of the appropriate level for tests and full reporting of outcomes
☒	☐	Estimates of effect sizes (e.g. Cohen's *d*, Pearson's *r*), indicating how they were calculated

Our web collection on statistics for biologists contains articles on many of the points above.

Software and code

Policy information about availability of computer code

Data collection	Roche LightCycler® 480 Software (Roche), BD FACSDivaTM software version 8.0 (BD), ZEN software (blue), (Zeiss), AxioVision 4.8 (Cytospin Slides imaging), Harmony High Content Imaging and Analysis Software 4.5 (Operetta, Perkin Elmer), HCS 3.4.0 Software for HiSeq 4000 Systems
Data analysis	FlowJo V10.2, Graph Pad Prism V6 (GraphPad Software), Columbus Image Data Storage and Analysis System (Operetta, Perkin Elmer), Ingenuity Pathway Analysis (Qiagen)

For manuscripts utilizing custom algorithms or software that are central to the research but not yet described in published literature, software must be made available to editors/reviewers. We strongly encourage code deposition in a community repository (e.g. GitHub). See the Nature Research guidelines for submitting code & software for further information.

Data

Policy information about availability of data

All manuscripts must include a data availability statement. This statement should provide the following information, where applicable:

- Accession codes, unique identifiers, or web links for publicly available datasets
- A list of figures that have associated raw data
- A description of any restrictions on data availability

RNA sequencing data has been deposited in NCBI's Gene Expression Omnibus and (GEO Series accession number GSE125150) (https://www.ncbi.nlm.nih.gov/geo/query/acc.cgi?acc=GSE125150).
Figures that are associated with the raw RNA sequencing date are Figure 4B, Supplementary Figure 7 and Supplementary Figure 11

Field-specific reporting

Please select the one below that is the best fit for your research. If you are not sure, read the appropriate sections before making your selection.

☒ Life sciences ☐ Behavioural & social sciences ☐ Ecological, evolutionary & environmental sciences

For a reference copy of the document with all sections, see nature.com/documents/nr-reporting-summary-flat.pdf

Life sciences study design

All studies must disclose on these points even when the disclosure is negative.

Sample size	Sample size for each experiment is indicated in the figure legend for each experiment. All experiments were conducted with cell lines with multiple available biological replicates and based on previous experience with specific experimental setup. Sample size was always determined to be adequate based on the magnitude and consistency of measurable differences between groups.
Data exclusions	No data were excluded from the analyses
Replication	All experiments were repeated so that our data are based on at least three biologically independent experiments with similar results. The precise number of repeats are stated in the figure legends.
Randomization	Not applicable, no animal studies reported in this manuscript.
Blinding	Investigators could not blinded to group allocation during experiments

Reporting for specific materials, systems and methods

We require information from authors about some types of materials, experimental systems and methods used in many studies. Here, indicate whether each material, system or method listed is relevant to your study. If you are not sure if a list item applies to your research, read the appropriate section before selecting a response.

Materials & experimental systems

n/a	Involved in the study	
☐	☒	Antibodies
☐	☒	Eukaryotic cell lines
☒	☐	Palaeontology
☒	☐	Animals and other organisms
☒	☐	Human research participants
☒	☐	Clinical data

Methods

n/a	Involved in the study	
☒	☐	ChIP-seq
☐	☒	Flow cytometry
☒	☐	MRI-based neuroimaging

Antibodies

Antibodies used	All antibodies for flow cytometry are stated in Table S1. Supplier, catalogue number and dilution used are included. For immunohistochemistry, it is stated in the METHODS section
Validation	Only certified and company-validated antibodies were purchased and used in the study.<br>Examples:<br>Anti-HA (1:500 dilution) (Clontech #631207) https://www.takarabio.com/assets/documents/Certificate%20of%20Analysis/631207-PA124303.pdf<br>25F9-efluor 660 (1:20 dilution)(ebioscience #15599866) https://www.fishersci.co.uk/shop/products/mature-macrophage-marker-mouse-anti-human-efluor-660-clone-ebio25f9-25f9-ebioscience-2/15599866<br>All antibodies were titrated to the lowest concentration in which the percentage of expressing cells was the same to the dilution suggested by the manufacturer

Eukaryotic cell lines

Policy information about cell lines

Cell line source(s)	Cell Line sources are clearly stated in the METHODS section
Authentication	The human iPSCs lines SFCi55 (parental, control) and SFCi55-iKLF1.2 were generated in house.The SFCi55 iPSC line was originally generated using fibroblasts obtained from blood group O Rhesus negative individuals by R Biomedical under REC 1/AL/0020 ethical approval and programmed to iPSCs using Yamanaka factors on episomal vectors. Both lines were confirmed to be pluripotent and have normal karyotype. SNP analyses were also carried out

	Frozen Umbilical cord blood (UCB) derived CD34+ cells were purchased from Stemcell Technologies (Cat No. 70008.5) from consenting donors with protocols approval by either the Food and Drug Administration (FDA) or an Institutional Review Board (IRB)
Mycoplasma contamination	All IPSC lines, and UCB CD34+ cells routinely tested negative for mycoplasma.
Commonly misidentified lines (See ICLAC register)	Not applicable

Flow Cytometry

Plots

Confirm that:

☒ The axis labels state the marker and fluorochrome used (e.g. CD4-FITC).

☒ The axis scales are clearly visible. Include numbers along axes only for bottom left plot of group (a 'group' is an analysis of identical markers).

☒ All plots are contour plots with outliers or pseudocolor plots.

☒ A numerical value for number of cells or percentage (with statistics) is provided.

Methodology

Sample preparation	Single cell suspensions were prepared using StemPro Accutase Cell Dissociation Reagent (Gibco) and re-suspended in PBS with 1%BSA and 5 mM EDTA. Cells were blocked with MACS FcR Blocking Reagent (#130-059-901) for 40 minutes on ice according to manufacturer instructions. 1x10^5 cell were washed and stained with appropriate antibodies (SupplementaryTable 1) for 20 minutes at room temperature. To assess enucleation, single cell suspensions were stained with Hoechst33342 1:20 (Thermofisher #R37605) for 20 min, washed with PBS 1%BSA and 5mM EDTA then stained with CD71-APC 1:200 (Thermofisher, 17-0719-42), CD235a-FITC 1:1000 (EBioscience #11-9987) and LIVE/DEADTM Fixable Near-IR Dead Cell Stain 1:100 (Thermofisher #L10119) for 20min at room temperature.<br>Cells were washed with PBS with 1%BSA and 5mM EDTA and kept on ice prior to analysis
Instrument	BD LSR Fortessa Analyzer (5 Laser)
Software	BD FACSDivaTM software version 8.0 (BD) was used to collect data and FlowJo V10.2 was used to analyse the data
Cell population abundance	At least 1x10^6 CD71-, Hoechst- cells were collected. Purity was confirmed by cytospin preparations<br>2X10^6 Hoechst+ cells were collected. Purity was confirmed by cytospin preparations<br>See supplementary Figure 2B
Gating strategy	Gating strategies are shown if Supplementary Figure S2A and supplementary Figure 3. In each experiment appropriate FMOs and positive controls were used to gate

☒ Tick this box to confirm that a figure exemplifying the gating strategy is provided in the Supplementary Information.
